# Draft Genome Sequences of Two *Lactobacillus casei* Strains Isolated from Cheddar Cheese and a Fermented Milk Drink

**DOI:** 10.1128/genomeA.01235-17

**Published:** 2017-11-02

**Authors:** Akhikun Nahar, Anthony L. Baker, John P. Bowman, Margaret L. Britz

**Affiliations:** a Tasmanian Institute of Agriculture, University of Tasmania, Hobart, Tasmania, Australia; b Faculty of Veterinary and Agricultural Sciences, The University of Melbourne, Melbourne, Victoria, Australia

## Abstract

MiSeq Illumina shotgun sequencing technology was used to sequence two *Lactobacillus casei* strains, designated strains GCRL 163 and MJA 12. The estimated genome sizes for GCRL 163 and MJA 12 were 2.9 Mb and 3.1 Mb, with 46.35% and 46.31% GC contents, respectively.

## GENOME ANNOUNCEMENT

The genus *Lactobacillus* within the phylum *Firmicutes* contains about 200 species that occupy diverse ecological niches, notably as endemic microflora of human and animal mucosal surfaces. Many species are of commercial importance due to their historical applications in the fermentation of plant, meat, and milk products, in the manufacture of bulk chemicals, and as probiotics and potential biodelivery agents (1). Members of the *L. casei* group, including species classified currently as *L. casei*, *L. paracasei*, and *L. rhamnosus*, are used as starter cultures in fermented milk products, and their presence in the nonstarter, adventitious microflora during ripening contributes to a cheese’s flavor (2). Of interest is how *L. casei* strains from different niches respond to and survive stresses encountered during manufacturing processes and probiotic consumption to confer functional traits (3). The full genome sequences of two *L. casei* strains, GCRL 163 (originally isolated from aged cheddar cheese) (4) and MJA 12 (isolated in this study from the fermented milk drink Yakult), are reported here.

Strains were grown in 1.5% glycine-supplemented MRS broth (Oxoid), cells were lysed by lysozyme/SDS treatment, and high-molecular-weight genomic DNA was extracted using modifications of a method originally described by Marmur (5). MiSeq Illumina technology was used to sequence the whole genomes (Macrogen Inc., Republic of South Korea). A total of 6,551,252 and 6,086,274 reads and 1,955,077,892 and 1,806,580,892 total read bases were obtained for *L. casei* GCRL 163 and MJA 12, respectively. ABySS (6) was used to assemble the whole-genome sequences. Based on 77 contigs (≥200 bp) the genome size of strain GCRL163 was 2.9 Mb with a GC content of 46.35%, and based on 96 contigs (≥200 bp) the genome size of strain MJA12 was 3.1 Mb with a GC content of 46.31%. The draft genome sequence was annotated using the NCBI Prokaryotic Genome Annotation Pipeline (7) and the Rapid Annotation using Subsystems Technology (RAST) server (8). A total of 2,938 genes and 2,807 coding genes for GCRL 163 and a total of 3,124 genes and 3,022 coding genes for MJA 12 were determined, with both strains showing >98.8% genetic similarity with *L. casei* ATCC 334 (Ga0029142) using the average nucleotide identity tool of the IMG database (9).

Using the RAST SEED Viewer, a total of 52 and 54 stress-response genes were found in the genomes of GCRL 163 and MJA 12, respectively, including 20 for oxidative stress and 14 for heat shock in both strains. 

Wuyts et al. (10) recently noted the ongoing and long-standing taxonomic inconsistencies in the nomenclature of strains currently classified as *L. casei* group members. They identified three possible clades in the *L. casei* group based on gene content in core orthogroups and predicted functional capacity. Based on the GC% content of the whole genomes, the detection of a manganese superoxide dismutase gene, the lack of catalase genes, and the failure to detect a SecA2/SecY2 protein secretion system clustered with glycosyltransferase genes, both GCRL 163 and MJA 12 would be classified as members of clade A, which currently comprises strains belonging to *L. casei* and *L. paracasei*. We detected multiple copies of genes annotated as *secA*, two copies of *yajC*, and single copies of *secE*, *secG*, and *secY*, none of which were obviously clustered together.

### Accession number(s).

These whole-genome shotgun projects have been submitted to DDBJ/EMBL/GenBank under the accession numbers MODT00000000 (GCRL 163) and MODS00000000 (MJA 12).
